# Semi‐Planar Non‐Fullerene Molecules Enhance the Durability of Flexible Perovskite Solar Cells

**DOI:** 10.1002/advs.202105739

**Published:** 2022-02-25

**Authors:** Hairui Liu, Zuhong Zhang, Zhenhuang Su, Weiwei Zuo, Ying Tang, Feng Yang, Xilin Zhang, Chaochao Qin, Jien Yang, Zhe Li, Meng Li

**Affiliations:** ^1^ School of Materials Science and Engineering Henan Normal University Xinxiang 453007 China; ^2^ Shanghai Synchrotron Radiation Facility (SSRF) Shanghai Advanced Research Institute Shanghai Institute of Applied Physics Chinese Academy of Sciences 239 Zhangheng Road Shanghai 201204 China; ^3^ Institute for Photovoltaics University of Stuttgart Pfaffenwaldring 47 Suttgart 70569 Germany; ^4^ Henan Key Laboratory of Photovoltaic Materials, School of Physics Henan Normal University Xinxiang 453007 China; ^5^ School of Engineering and Materials Science (SEMS) Queen Mary University of London London E1 4NS UK; ^6^ Key Lab for Special Functional Materials Ministry of Education National & Local Joint Engineering Research Center for High‐Efficiency Display and Lighting Technology School of Materials Science and Engineering and Collaborative Innovation Center of Nano Functional Materials and Applications Henan University Kaifeng 475004 China

**Keywords:** flexible perovskites, mechanical stability, non‐fullerene, semiconductors, SnO_2_, stress

## Abstract

Flexible perovskite solar cells (FPSCs) represent a promising technology in the development of next‐generation photovoltaic and optoelectronic devices. SnO_2_ electron transport layers (ETL) typically undergo significant cracking during the bending process of FPSCs, which can significantly compromise their charge transport properties. Herein, the semi‐planar non‐fullerene acceptor molecule Y6 (BT‐core‐based fused‐unit dithienothiophen [3,2‐b]‐pyrrolobenzothiadiazole derivative) is introduced as the buffer layer for SnO_2_‐based FPSCs. It is found that the Y6 buffer layer can enhance the ability of charge extraction and bending stability for SnO_2_ ETL. Moreover, the internal stress of perovskite films is also reduced. As a result, SnO_2_/Y6‐based FPSCs achieved a power conversion efficiency (PCE) of 20.09% and retained over 80% of their initial efficiency after 1000 bending cycles at a curvature radius of 8 mm, while SnO_2_‐based devices only retain 60% of their initial PCE (18.60%) upon the same bending cycles. In addition, the interfacial charge extraction is also effectively improved in conjunction with reduced defect density upon incorporation of Y6 on the SnO_2_ ETL, as revealed by femtosecond transient absorption (Fs‐TA) measurements.

## Graphical abstract

Y6 passivation layer can enhance SnO_2_ ETL durability during bending processes. The SnO_2_/Y6‐based FPSCs achieve a PCEof 20.09% and retain over 80% of their initial efficiency after 1000 bending cycles at a curvature radius of 8 mm, while SnO_2_‐based devices only retain 60% of their initial PCE (18.60%) upon the same bending cycles.

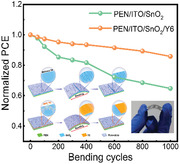

## 1 Introduction

Organic–inorganic hybrid perovskite solar cells (PSCs) have attracted significant attention owing to the superior optoelectronic properties of perovskite materials such as low trap density, suitable bandgap, and long charge‐carrier diffusion lengths.^[^ ^1^ ^]^ Significant effort has been dedicated to the optimization of their materials and device design, resulting in a rapid increase of theirPCE from 3.8% in 2009^[^ ^2^ ^]^ to 25.5% in 2021.^[^ ^3^ ^]^ These efforts can be broadly divided into three main categories, namely crystallization control,^[^ ^4^ ^]^ composition modulation,^[^ ^5^ ^]^ and interface engineering.^[^ ^6^ ^]^ In particular, interface engineering has been established as an effective approach for improving the device performance of PSCs, especially for FPSCs where internal stresses are accentuated. And SnO_2_ has been identified as one of the most widely used ETLs in PSCs, owing to their good electron mobility, high transmittance, smaller refractive index, and ease of low‐temperature processing.^[^ ^7^ ^]^ However, SnO_2_ still faces several shortcomings for use in PSCs. For example, the growth of organic and inorganic perovskite films on SnO_2_ ETL usually undergoes a process of rapid heating and cooling. The smaller thermal expansion and cooling contraction coefficient of the SnO_2_ ETL than that of the perovskite absorber can result in the creation of residual stress inside the perovskite crystals, which can seriously deteriorate the stability of perovskite films.^[^ ^8^ ^]^ To overcome this issue, many methods have been developed to release the residual stress in PSCs,^[^ ^8^, ^9^ ^]^ including optimization of the thermal annealing process,^[^ ^9b,e^ ^]^ interface post‐treatment^[^ ^9a,c^ ^]^ and composition alloying.^[^ ^9d^ ^]^

Furthermore, significant effort has also been dedicated to the performance optimization of FPSCs based on SnO_2_ ETL. Chung et al. introduced the ZnSO_4_ porous layer to maximize the charge collection efficiency and minimize the carrier recombination losses in FPSCs.^[^ ^10^ ^]^ Huang et al. studied the effect of morphology of different concentrations of SnO_2_ on the stability of flexible devices.^[^ ^11^ ^]^ Zhou et al. introduced fullerene derivatives into the interface between SnO_2_ and perovskite film to increase the device's durability against mechanical bending.^[^ ^12^ ^]^ Although the FPSCs based on SnO_2_ ETL performance were improved by some strategies, the obstacles that hinder the performance of flexible devices also need to be overcome. For example, SnO_2_ on the flexible substrate suffers from serious cracks after bending, causing PCE degradation due to these cracks to exist as recombination centers that result in severe nonradiative recombination.

In this work, we introduce the semi‐planar organic non‐fullerene small molecule Y6 as a buffer layer between the perovskite photoactive layer (Cs_0.05_(FA_0.87_MA_0.13_)_0.95_Pb(I_0.87_Br_0.13_)_3_) and SnO_2_ ETL to simultaneously release the residual stress created during the thermal annealing process and reduce the formation of defects against mechanical bending. It is found that Y6 act as a buffer layer that possesses optimal binding energy, with a difference *q* value of 0.034 nm^−1^ (a difference *q* value of 0.059 nm^−1^ for SnO_2_‐based samples) between out‐of‐plane and in‐plane orientation during the thermal annealing process, resulting in relieving stress and enhanced stability of the perovskite photoactive layer. As a result, substantially reduced cracking is observed both in the SnO_2_ ETL and perovskite photoactive layer employing a Y6 buffer layer compared to SnO_2_‐based films without a Y6 buffer layer upon mechanical bending. The Young's modulus is found to decrease from 3920 MPa in SnO_2_‐based perovskite films to 3090 MPa in SnO_2_/Y6‐based perovskite films, indicating that Y6 can effectively decrease the bending resistance of the perovskite films. Fs‐TA measurements reveal that the Y6 buffer layer can effectively enhance the interfacial charge transport rate, evidenced by a substantially reduced delay time from 24.67 ns to 10.09 ns through dynamics fitting, indicating excellent electron transport properties. As a result, FPSCs employing a Y6 buffer layer not only exhibit superior device performance (with a PCE of 20.09% versus PCE of 18.60% in SnO_2_‐based devices), but also maintain 80% of their initial PCE after 1000 mechanical bending cycles, while SnO_2_‐based FPSCs only maintain 60% of their initial PCE value. We further compared the conventional fullerene PCBM molecule with the excellent non‐fullerene Y6 molecule and proved the excellent properties of non‐fullerenes in improving SnO_2_ ETL robustness during the bending process. So, the incorporation of a buffer layer based on non‐fullerene organic semiconductors is a promising route for the simultaneous enhancement of device performance and mechanical robustness of FPSCs.

## 2 Results and Discussion

**Figure 1:**
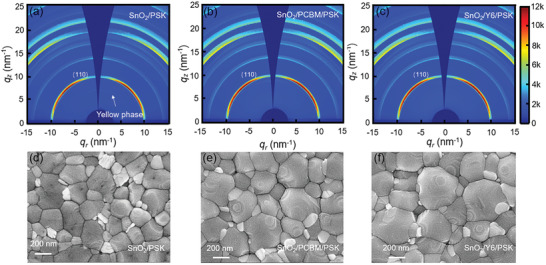
a–c) GIXRD and d–f) SEM images of perovskite films grow on SnO_2_ ETL, SnO_2_/PCBM, and SnO_2_/Y6 substrate.

The under layers play an important role in the perovskite crystallization kinetics and thus the performance of PSCs. **Figure** **1** compares the perovskite films morphology and crystal characteristics on different substrates. 2D grazing incidence X‐ray diffraction (GIXRD) was carried out to investigate the perovskite films crystal characteristics. Figure 1a–c shows different scattering patterns about SnO_2_‐based, SnO_2_/PCBM‐based, and SnO_2_/Y6‐based perovskite films, respectively. The intensity of peak along (110) scattering ring in SnO_2_/Y6‐based perovskite films is stronger than that of SnO_2_‐based and SnO_2_/PCBM‐based perovskite films, indicating the greatest level of crystallization of the perovskite materials, which is further confirmed as illustrated in Figure S1 (Supporting Information). Perovskite films deposited on SnO_2_ ETL exhibit the poorest crystallization characteristics, with a yellow phase scattering ring in the GIXRD image. This is further confirmed by X‐ray diffraction (XRD) studies, with the perovskite films deposited on 0.75 mg mL^−1^ Y6 exhibiting the highest diffracted intensity at (110) crystal planes, while yellow phase is also observed for the SnO_2_‐based perovskite films. In comparison, the (110) crystal planes peak intensities of SnO_2_/PCBM‐based perovskite films are lower than that of SnO_2_/Y6‐based perovskite films (Figure S2, Supporting Information). These results indicate that introducing the Y6 buffer layer has a significant influence on the crystallization of perovskite films, and it can effectively prevent the formation of the yellow phase.

The influence of different underlayers on perovskite crystals morphology was investigated by the field emission scanning electron microscope (SEM), as shown in Figure 1d–f. A high population of pinholes is observed in perovskite films deposited on neat SnO_2_ ETL. It is widely recognized that pinholes can act as recombination centers, resulting in reduced carriers’ quantity arriving in the electrodes. In contrast, both Y6 and PCBM modified SnO_2_ ETL due to Y6 and PCBM provide conjugated double bonds that can significantly improve the growth of perovskite crystals, with the perovskite films on the SnO_2_/Y6 substrate possessing the largest grain size. Statistical histograms (Figure S3, Supporting Information) show that the mean grain size of the perovskite crystals is 198, 259, and 285 nm for perovskite films deposited on SnO_2_ ETL, SnO_2_/PCBM, and SnO_2_/Y6 substrate, respectively. The morphology of perovskite films grown on different concentrations of Y6 modification SnO_2_ ETL shows that 0.75 mg mL^−1^ Y6 is in favor of enhancing perovskite crystal size. (Figure S4, Supporting Information).

**Figure 2:**
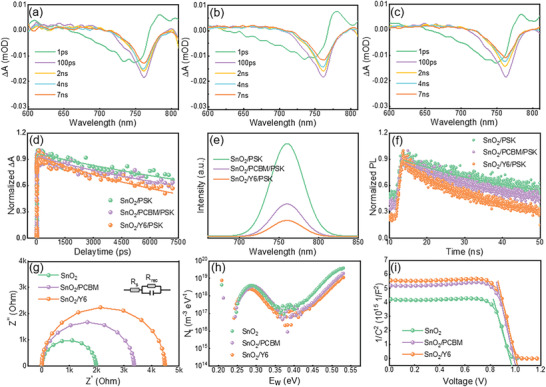
TAS of perovskite films grown on a) SnO_2_, b) SnO_2_/PCBM, c) SnO_2_/Y6, d) Normalized bleaching kinetics for perovskite films grow on SnO_2_, SnO_2_/PCBM, and SnO_2_/Y6 at 760 nm e) PL and f) TRPL spectra of perovskite films deposited on SnO_2_ and SnO_2_/Y6 g) Nyquist plots of PSCs measured at a bias of 1 V h) The t‐DOS characteristics i) Mott–Schottky characteristic results.

To study the characteristics of the carrier transport dynamics, we conducted Fs‐TA measurement. Perovskite films based on different substrates were photoexcited at a wavelength of 365 nm within the wavelength range of 600–800 nm, and the absorption peak was ≈760 nm. **Figure** **2**a–c shows the transient absorption spectrum (TAS) of SnO_2_‐based, SnO_2_/PCBM‐based, and SnO_2_/Y6‐based samples. The negative peak at ≈760 nm indicates the electron transition from low energy to high energy levels. After the sample absorbs the pump light, electrons transit to an excited state, which reduces the number of electrons in the ground state. The ground state absorption of the sample in the excited state is less than the ground state absorption of the unexcited sample, and a negative Δ*A* signal is detected. As the delay time increases, a faster decay of peak intensity is observed in SnO_2_/Y6‐based samples than SnO_2_‐based and SnO_2_/PCBM‐based samples, indicating more efficient carrier transport resulting in the shortest exciton lifetime of the SnO_2_/Y6‐based samples.^[^ ^13^ ^]^

The decay kinetics was extracted at the bleaching wavelength of ≈760 nm. As shown in Figure 2d, the fitting values of *τ*_2_ decreased from 24.67 ns (SnO_2_‐based samples) to 10.09 ns (SnO_2_/Y6‐based samples). These results show that charge extraction is faster at the SnO_2_/Y6‐based samples. The *τ*_1_ and *τ*_2_ of the three samples are listed in Table S1 (Supporting Information). Steady‐state photoluminescence (PL) and time‐resolved PL (TRPL) spectra were measured to explore the trap density and charge transport properties between the perovskite films and ETLs. As shown in Figure 2e, the PL peaks of SnO_2_/Y6‐based, SnO_2_/PCBM‐based, and SnO_2_‐based samples are located at ≈760 nm. The low peak intensity of SnO_2_/Y6‐based samples indicates excellent electrons transfer due to intimate contact between the perovskite material and SnO_2_/Y6 substrate.^[^ ^14^ ^]^ TRPL spectra are shown in Figure 2f, and the fitting values of *τ*_2_ are 35.26, 50.12, and 150.46 ns for SnO_2_/Y6‐based, SnO_2_/PCBM‐based, and SnO_2_‐based samples, respectively. SnO_2_/Y6‐based samples show the shortest PL lifetime, indicating the fastest charge extraction and lowest recombination rate during the carrier transport process among the perovskite films based on the three ETLs.^[^ ^15^ ^]^

Electrochemical impedance spectroscopy (EIS) measurement was performed to characterize the charge carrier recombination at open‐voltage (≈1 V), series resistance (*R*_s_), and recombination resistance (*R*_rec_) of different PSCs employing different ETLs, which can be obtained from the Nyquist plots intuitively. The equivalent circuits are presented in Figure 2g. The semi‐circle of the SnO_2_/Y6‐based devices is larger than that of the SnO_2_/PCBM‐based and SnO_2_‐based devices, indicating a significant reduction in charge carrier recombination rate, attributed to the lower *R*_s_ and higher *R*_rec_.^[^ ^16^ ^]^

We further undertake trap density of states (t‐DOS) measurements to characterize the state distribution of complete devices. As shown in Figure 2h, the t‐DOS in the shallow trap region of SnO_2_/Y6‐based devices is lower than SnO_2_/PCBM‐based and SnO_2_‐based devices. This means that the introduction of the Y6 buffer layer can effectively suppress trap formation at grain boundaries and interface.^[^ ^17^ ^]^

The Mott–Schottky curve was tested to obtain the value of *V*_bi_, which is strongly correlated to the separation of the photogenerated carriers.^[^ ^18^ ^]^ As shown in Figure 2i, the *V*_bi_ are 1.01, 0.99, and 0.97 eV for the SnO_2_/Y6‐based, SnO_2_/PCBM‐based, and SnO_2_‐based devices, respectively. The greater *V*_bi_ of SnO_2_/Y6‐based devices indicates a more efficient separation of photogenerated carriers.^[^ ^19^ ^]^

**Figure 3:**
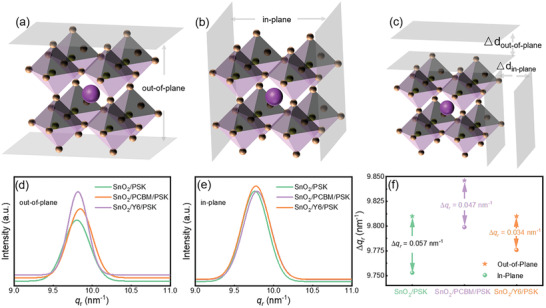
Schematic architecture of the a) out‐of‐plane, and b) in‐plane lattice orientation. c) Equivalent diagram of perovskite lattice shrinkage in out‐of‐plane and in‐plane directions. The *q* value of d) out‐of‐plane, and e) in‐plane lattice orientation. f) Schematic diagram of *q* value difference between out‐of‐plane and in‐plane lattice orientation.

To investigate the internal residual stress due to lattice expansion and constriction in perovskite films deposited on different substrates, the lattice change results of out‐of‐plane and in‐plane orientation about three perovskite films were carried out. As shown in **Figure** **3**a,b, the orientation of an out‐of‐plane is vertical to the unit cell underside, that of an in‐plane is parallel to the unit cell underside. The change of *q* value (where *q* is the scattering vector and calculated using the formula *q* = 4*π*sin(*θ*)/*λ*, where *θ* is the incident ray, the angle between the reflected line and the reflective crystal plane, *λ* a is the incident ray) along the out‐of‐plane and in‐plane orientation is caused by the lattice expansion and constriction (Figure 3c).^[^ ^20^ ^]^ The smaller difference of *q* value between out‐of‐plane and in‐plane orientation, the smaller the residual stress in the films. The calculated *q* value difference (Δ*q*) between the out‐of‐plane (Figure 3d) and in‐plane (Figure 3e) orientation of SnO_2_‐based_,_ SnO_2_/PCBM‐based, and SnO_2_/Y6‐based samples are 0.057, 0.047, and 0.034 nm^−1^ (Figure 3f), respectively. SnO_2_ as a metal oxide has a smaller thermal expansion and cooling constriction coefficient than the perovskite materials. During the heating and cooling process, the expansion and constriction of the perovskite crystal lattice in the in‐plane orientation is smaller than that in the out‐of‐plane orientation, leading to a larger Δ*q* value. While organic molecules, especially Y6, have a certain degree of robustness and less restriction on lattice deformation, which can effectively reduce the generation of stress. We further performed a simulation of the binding energy between the perovskite crystal and the underlayer. As shown in Figure S5 (Supporting Information), the large binding energy (‐15.79 eV) between the perovskite crystal and SnO_2_ can cause the fracture of the crystal lattice due to severe lattice distortion, while much lower binding energy between perovskite crystal and Y6 is calculated at −5.77 eV, indicating that the Y6 buffer layer can effectively decrease the binding energy of the layers to release the residual stress generated due to lattice distortion.

**Figure 4:**
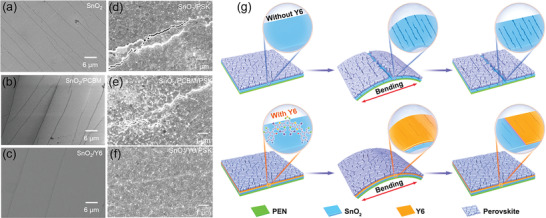
SEM images of a) SnO_2_, b) SnO_2_/PCBM, c) SnO_2_/Y6, d) Control perovskite, e) PCBM‐based perovskite, and f) Y6‐based perovskite after 1000 bending cycles at a curvature radius of 8 mm. g) Schematic diagram of Y6 action mechanism.

To assay the effect of bending on SnO_2_ ETL and perovskite films, we investigate the morphological changes of both SnO_2_ ETLs, and perovskite films after 1000 bending cycles with a curvature radius of 8 mm. As shown in **Figure** **4**a,b, significant cracking is observed on the surface of pristine SnO_2_ ETL and SnO_2_/PCBM substrate caused by the strong mechanical stress. In contrast, fewer cracks are seen on SnO_2_/Y6 surface (Figure 4c), indicating that the Y6 layer can effectively reduce cracking of the SnO_2_ ETL during the bending process, leading to minimal compromise in the ETL performance. As shown in Figure 4d,e, large cracks are seen on perovskite films based on SnO_2_ ETL and SnO_2_/PCBM substrates due to bending, which can lead to serious current leakage and carrier recombination. In contrast, minimal cracking can be observed for perovskite films based on SnO_2_/Y6 substrates (Figure 4f), indicating that the Y6 buffer layer also enhances the robustness of perovskite films. Figure 4g illustrates the schematic diagram of the formation of cracks of perovskite films based on different flexible substrates induced by bending.

**Figure 5:**
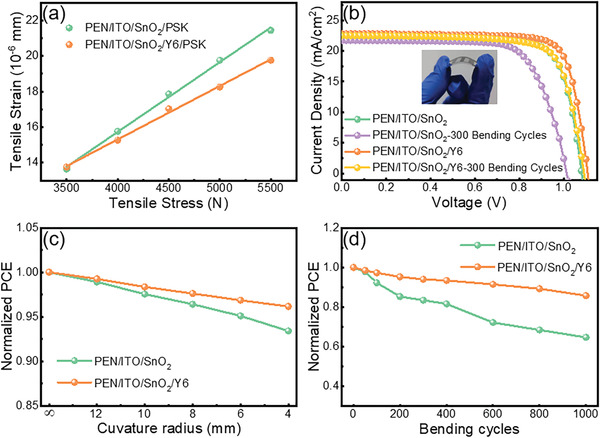
a) Tensile strain of perovskite films on SnO_2_ and SnO_2_/Y6 under different tensile stress. b) The *J–V* curves of FPSCs based on SnO_2_ and SnO_2_/Y6, separately. c) PCE changes of FPSCs after bending different curvature radius. d) PCEs of FPSCs as a function of bending cycles at a bending curvature radius of 8 mm.

To explore the relationship between the internal tensile strain and tensile stress of the perovskite films based on different substrates, we use a universal testing machine to test the change of strain under different tensile strengths. The results are shown in **Figure** **5**a. The Young's modulus of perovskite films on SnO_2_ and SnO_2_/Y6 are 3920 and 3090 MPa, respectively. Under the same tension, perovskite films grown on SnO_2_/Y6 have a larger strain space, indicating higher compatibility with flexible substrates, thus rationalizing why Y6‐based perovskite films possess a higher level of robustness.

The photoelectric properties of PSCs based on different flexible substrates are further studied, and the *J–V* curves are shown in Figure 5b. The PCE of FPSCs based on SnO_2_/Y6 substrate and SnO_2_ ETL were 20.09% and 18.60%, which is decreased to 18.42% and 15.76% after 300 bending cycles at the curvature radius of 8 mm. The corresponding *V_OC_*, *J_SC,_* and FF are listed in Table S3 (Supporting Information). To further study the bending resistance of the devices, we tested the PCE of the flexible devices at different curvature radius (Figure 5c). Rapid degradation is seen in the PCE of SnO_2_‐based devices after bending at a large curvature radius, suggesting severe internal damage of the SnO_2_‐based devices after bending tests. In contrast, significantly slower degradation is seen in the SnO_2_/Y6‐based devices. The corresponding devices parameters are shown in Figure S6 (Supporting Information). The fatigue test was carried out by bending the devices 1000 times at a curvature radius of 8 mm, as shown in Figure 5d and S7 (Supporting Information). The PCE of SnO_2_‐based devices was reduced to 60% of the initial value, while the SnO_2_/Y6‐based devices could maintain ≥80% original PCE. Although SnO_2_/Y6‐based devices are damaged to a certain extent, the PCE is mostly retained compared with the SnO_2_‐based devices, indicating Y6 buffer layer can efficiently enhance device robustness.

For a more comprehensive study, we also fabricated devices on rigid ITO glass. Figure S8 (Supporting Information) illustrates *J–V* curves of PSCs based on SnO_2_ ETL, SnO_2_/PCBM (the optimized concentration of PCBM was 2 mg ml^−1^, Figure S9, Supporting Information), SnO_2_/Y6 (the optimized concentration of Y6 was 0.75 mg ml^−1^, Figure S10, Supporting Information) substrate. The best devices exhibit a PCE of 20.12% on pristine SnO_2_ ETL, 20.98% on SnO_2_/PCBM substrate, and 22.19% on SnO_2_/Y6 substrate. The parameters of the above devices are summarized in Table S4 (Supporting Information).

The hysteresis index (HI) as a critical evaluation parameter for device quality was assessed, as shown in Figure S11 (Supporting Information). The HI value of the SnO_2_‐based devices (0.04) is twice that of SnO_2_/Y6‐based devices (0.02), so the SnO_2_/Y6‐based devices exhibit negligible hysteresis than others. The small HI of SnO_2_/Y6‐based devices indicates favorable contact between the perovskite layer and ETL.^[^ ^21^ ^]^ To test the device's operational stability, the stabilized PCE versus time under one sun irradiation was carried out. The SnO_2_/Y6‐based devices can maintain 20.45% PCE for 400 s at maximum power point (MPP). However, the PCE of SnO_2_/PCBM‐based and SnO_2_‐based devices can only retain 19.64% and 18.34% (Figure S12, Supporting Information). The *J_SC_* calculated from the incident‐photon‐to‐current efficiency (IPCE) curves of devices based on different substrates (Figure S13, Supporting Information) are 22.42 (SnO_2_‐based devices), 23.01 (SnO_2_/PCBM‐based devices), and 23.26 (SnO_2_/Y6‐based devices) mA cm^−2,^ respectively, which matches well with the *J–V* measurement results under AM 1.5G single sunlight exposure. The stability tests of all devices were also conducted under continuous illumination. As shown in Figure S14a (Supporting Information), the SnO_2_/Y6‐based devices can maintain 79.82% of the initial PCE after 600 h. The SnO_2_/PCBM‐based and SnO_2_‐based devices only maintain 69.11% and 40.12%, respectively, which may be related to the crystallinity of the perovskite films. The decay trend of *V_OC_*, *J_SC,_* and FF is shown in Figure S14b–d (Supporting Information).

## 3 Conclusion

We investigated the impact of the processing of SnO_2_ ETL on the performance and durability of FPSCs. It is found that the introduction of the Y6 buffer layer can substantially reduce the formation of cracks of SnO_2_ on flexible substrates during the bending process and relieve the residual stress of perovskite films during the crystallization process, which results in not only improved device performance, but also substantially enhanced durability under mechanical bending and light soaking stress. Our findings indicate that the incorporation of a buffer layer based on non‐fullerene organic semiconductors is a promising route for the simultaneous enhancement of device performance and mechanical robustness of FPSCs.

## Conflict of Interest

The authors declare no conflict of interest.

## Authors Contribution

H.L. and Z.Z. contributed equally to this work. H.L., Z.Z., M.L., and Z.L. designed and discussed the experiments for this work and wrote the manuscript. H.L. and M.L. guided this work. F.Y., Z.L., W.Z., and J.Y. modified the manuscript. Z.Z. and Y.T. fabricated and characterized the perovskite solar cells and films. Z.S. conducted the GIXRD test. C.Q. carried out the fs‐TA measurements. X.Z. calculated the binding energy simulation. All authors contributed to the writing of the paper.

## Supporting information

Supporting Information

## Data Availability

The data that support the findings of this study are available from the corresponding author upon reasonable request.
