# Supplementary material for: Semi‐Planar Non‐Fullerene Molecules Enhance the Durability of Flexible Perovskite Solar Cells
Source: Adv Sci (Weinh). 2022 Feb 25;9(11):2105739. doi: 10.1002/advs.202105739 (PMC9008411; doi:10.1002/advs.202105739)
Supplement: Supplementary file 1 — Supporting Information [file ADVS-9-2105739-s001.pdf]

ADVANCED SCIENCE
Open Access

Supporting Information

for *Adv. Sci.*, DOI 10.1002/advs.202105739

Semi-Planar Non-Fullerene Molecules Enhance the Durability of Flexible Perovskite Solar Cells

Hairui Liu, Zuhong Zhang, Zhenhuang Su, Weiwei Zuo, Ying Tang, Feng Yang, Xilin Zhang, Chaochao Qin, Jien Yang, Zhe Li* and Meng Li**

Supporting Information

Semi-Planar Non-Fullerene Molecules Enhance the Durability of Flexible Perovskite Solar Cells

Hairui Liu, Zuhong Zhang, Zhenhuang Su, Weiwei Zuo, Ying Tang, Feng Yang, Xilin Zhang, Chaochao Qin, Jien Yang, Zhe Li*, Meng Li**

Dr. H. Liu, Z. Zhang, Dr. J. Yang
School of Materials Science and Engineering,
Henan Normal University,
Xinxiang 453007, China

Prof. M. Li
Key Lab for Special Functional Materials, Ministry of Education, National & Local Joint Engineering Research Center for High-efficiency Display and Lighting Technology, School of Materials Science and Engineering, and Collaborative Innovation Center of Nano Functional Materials and Applications,
Henan University,
Kaifeng 475004, China
E-mail: mengli@henu.deu.cn

Dr. Z. Su
Shanghai Synchrotron Radiation Facility (SSRF), Shanghai Advanced Research Institute, Shanghai Institute of Applied Physics Chinese Academy of Sciences,
239 Zhangheng Road, Shanghai 201204, China

W. Zuo
Institute for Photovoltaics,
University of Stuttgart,
Pfaffenwaldring 47, 70569 Suttgart, Germany
E-mail: weezuo@gmail.com

Y. Tang, Dr. X. Zhang, Dr. F. Yang, Dr. C. Qin
Henan Laboratory of Photovoltaic Materials, School of physics,
Henan Normal University,
Xinxiang 453007, China

Prof. Z. Li
School of Engineering and Materials Science (SEMS),
Queen Mary University of London,
London, E1 4NS UK
E-mail: zhe.li@qmul.ac.uk

Materials preparation: Formamidinium iodide (FAI, ≥99.5%), Methylammonium bromide (MABr, 99.5%), Lead bromide ($PbBr_2$, 99.9%), Cesium Iodide (CsI, 99.9%), [6,6]-phenyl-C61-butyric acid methyl ester ($PC_{61}BM$, 99%), 2,2',7,7'-Tetrakis [N, N-di(4-methoxyphenyl) amino]-9,9'-spirobifluorene (spiro-OMeTAD, 99.8%), lithium bistrifluorosulfonyl imide (Li-TFSI, ＞99%) and 4-tert-butyl pyridine (t-BP, ＞96%) were purchased from Xi'an Polymer Light Technology. Y6 was purchased from Solarmer Material Inc. Dimethyl sulfoxide (DMSO, ＞99.7%), dimethylformamide (DMF, 99.5%), chlorobenzene (CB, 99.5%) and isopropanol (IPA, 99.9%) were purchased from Sigma-Aldrich. SnO_2 colloidal dispersion (tin (IV) oxide, 15% in H_2O) and ultra-pure water was purchased from Alfa Aesar.

Device Fabrication: ITO glass was cleaned sequentially with detergent water, deionized (DI) water, acetone, and ethanol for 15 minutes in ultrasonic system. The SnO_2 (SnO_2/water=1:4 v/v) layer was deposited on ITO substrates at 3000rpm for 30 s. After that, the substrates were annealed at 150°C for 30 minutes to improve the electrical properties. After UV treatment, the ITO substrates were transferred into glove box. 100 μL of perovskite solution was spin-coated on SnO_2 layer at 1000 rpm for 10 s and 6000 rpm for 30s (172 mg FAI, 507 mg PbI_2, 16.8 mg MABr and 60.6 mg $PbBr_2$ in 1ml DMF/DMSO=4:1, v/v, then 50 μL 1.5 mol/L CsI DMSO solution added into perovskite solution), the film was annealed at 100°C for 40 minutes. Spiro-OMeTAD (90 mg) was dissolved in 1 mL chlorobenzene mixed with 22.5 μL Li-TFSI (520 mg/mL in acetonitrile) and 36 μL 4-tert-butylpyridine (t-BP). The

Spiro-OMeTAD was deposited at speed of 5000 rpm for 40 s. Finally, 10 nm MoO_3 and100 nm Au was deposited by thermal evaporation system. Every cell size is 0.18 cm^2.

SnO_2 ETL Treatment: The 100 ml 3% SnO_2 colloidal dispersion (It was diluted using DI) was spin-coated on cleaning ITO for 3000 rpm 30s and then the ITO was annealed at 150℃ for 30 minutes. The different concentration Y6 and PCBM CB solutions were spin-coated on SnO_2 ETL for 2000 rpm 30 s and then the treatment SnO_2 ETLs were annealed at 100 ℃ for 5 minutes.

Flexible Device Fabrication: Fix the flexible PEN substrate on the transparent glass. The next steps are the same as the rigid substrate.

Characterization: X-ray diffraction (XRD) (Rigaku D/MAX-2400 diffractometer) and field emission scanning electron microscope (SEM) (FESEM, Zeiss Supra55) (Bruker, Multi-Mode 8) and the grazing incidence X-ray diffraction (GIXRD) (BL14B1 beamline of the Shanghai Synchrotron Radiation Facility (SSRF) using X-ray with a wavelength of 1.24 Å) were used for characterizing the crystal characterization of perovskite film. J-V curves of complete devices were measuring by Sunlight Simulator for Taiwan enlitechnology (KA5000) and a digital source meter Keithley 2400. The cells were masked to give defined active area of 0.18 cm^2. The incident-photon-to-current efficiency (IPCE) was tested by QE-R of Taiwan enlitechnology. Steady-state photoluminescence (PL) and time resolved photoluminescence (TRPL) spectra were tested by Steady state transient fluorescence spectrometer with a 460 nm pulsed laser (Edinburghinstruments, FLS980). Helios

pump-probe system (Ultrafast Systems LLC) coupled with an amplified femtosecond laser system (Coherent, 35 fs, 1 kHz, 800 nm) were used for measuring Femtosecond transient absorption (fs-TA) measurements. Electrical Impedance Spectroscopy (EIS) (1 V bias voltage, 10 HZ-1 MHZ) and Mott–Schottky (The tested frequency was 1000 Hz) carried out using an Ivium Electrochemical Workstation (Netherlands) under dark conditions. T-DOS results were tested using a Precision Impedance Analyzer (Agilent 4294A, 40 HZ-1 MHZ).

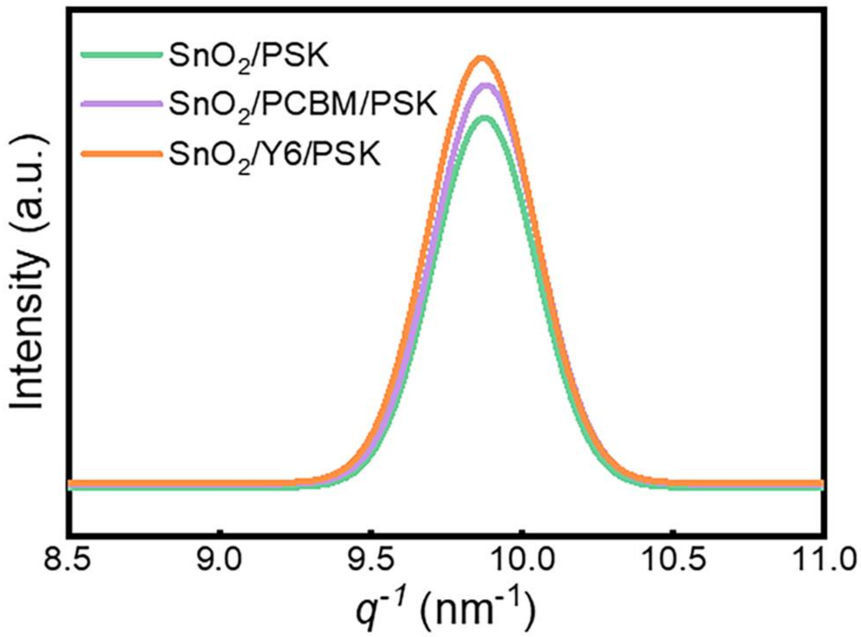

Figure S1 Derived 1D GIXRD spectra along (110) diffraction ring.

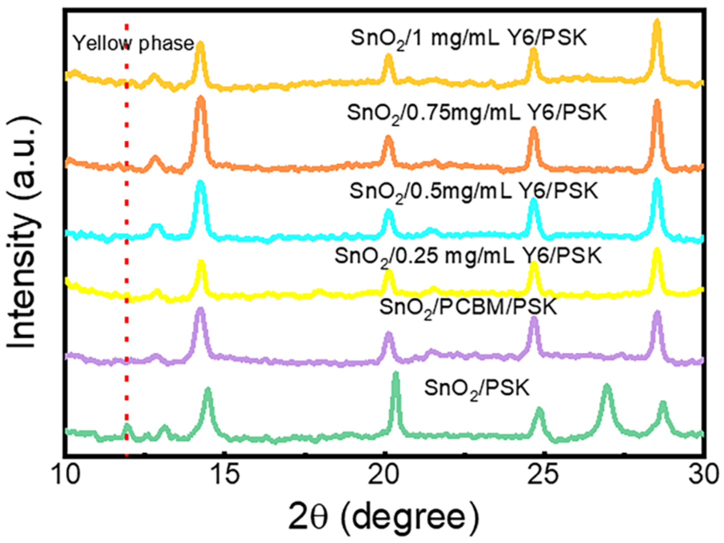

Figure S2. XRD patterns of perovskite on different substrate.

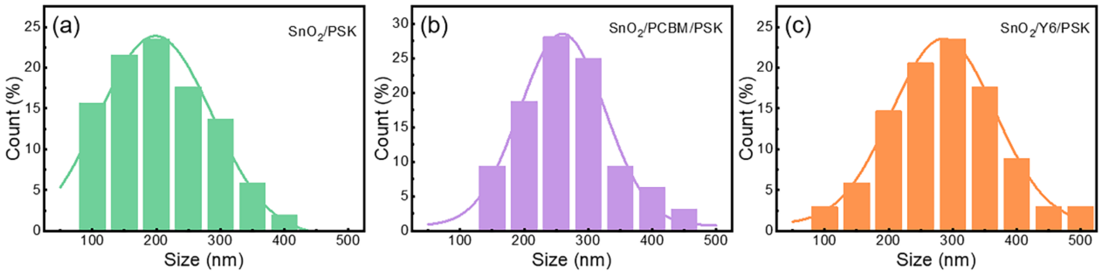

Figure S3. Statistics histogram of perovskite grain on a) SnO_2 b) SnO_2/PCBM c) SnO_2/Y6.

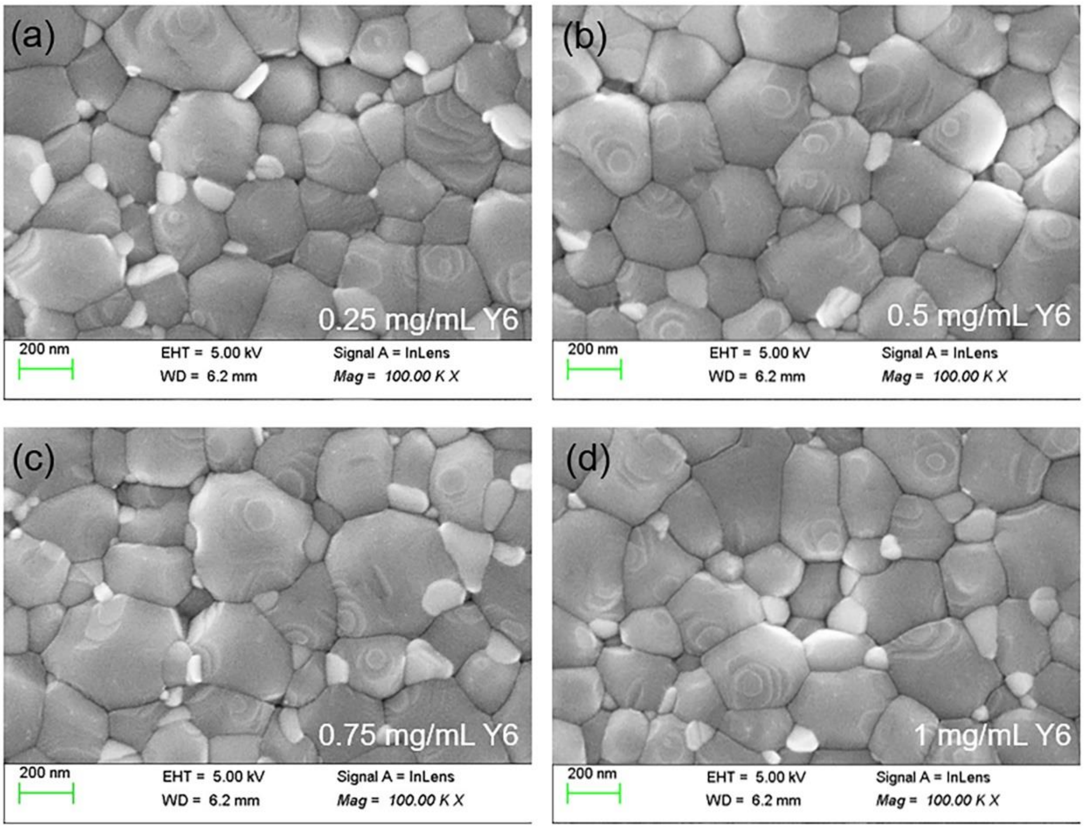

Figure S4. SEM images of perovskite on a) SnO_2/0.25mg/mL Y6 b) SnO_2/0. 5mg/mL Y6 c) SnO_2/0.75mg/mL Y6 d) SnO_2/1mg/mL Y6.

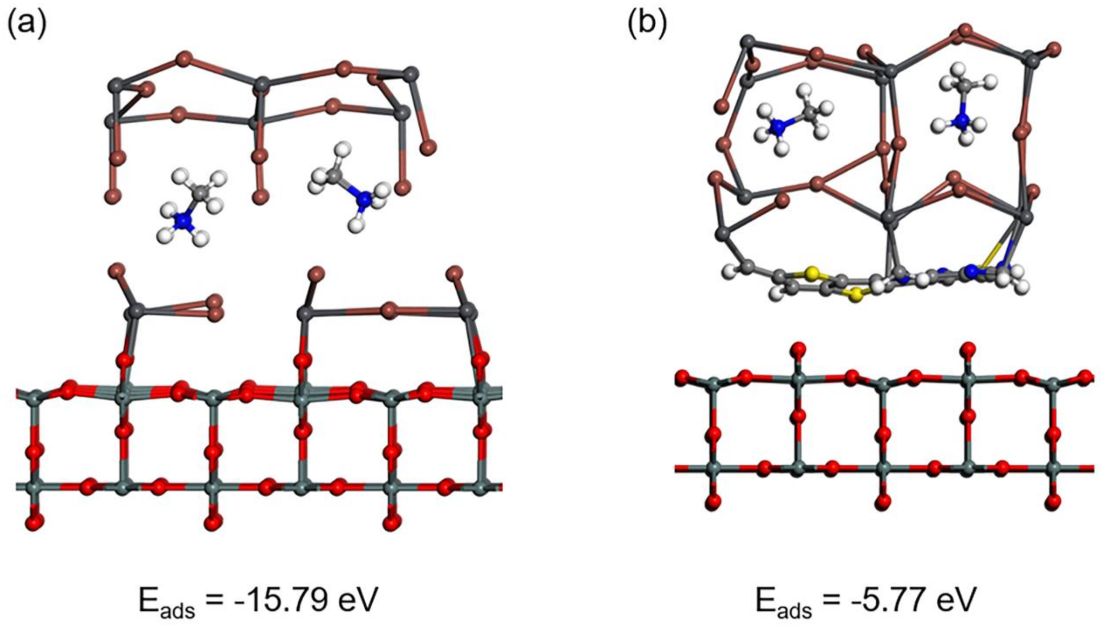

Figure S5. Calculated binding energy of perovskite and a) SnO_2 b) Y6.

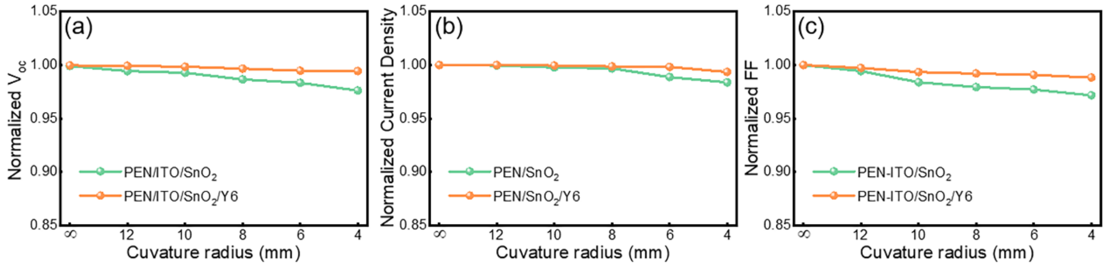

Figure S6. a) V_{OC} b) J_{SC} c) FF changes of FPSCs after bending different curvature radius.

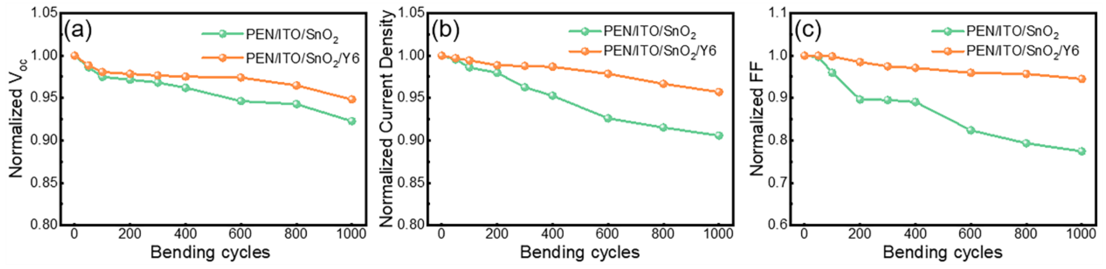

Figure S7. a) V_{OC} b) J_{SC} c) FF of FPSCs as a function of bending cycles at a curvature radius of 8 mm.

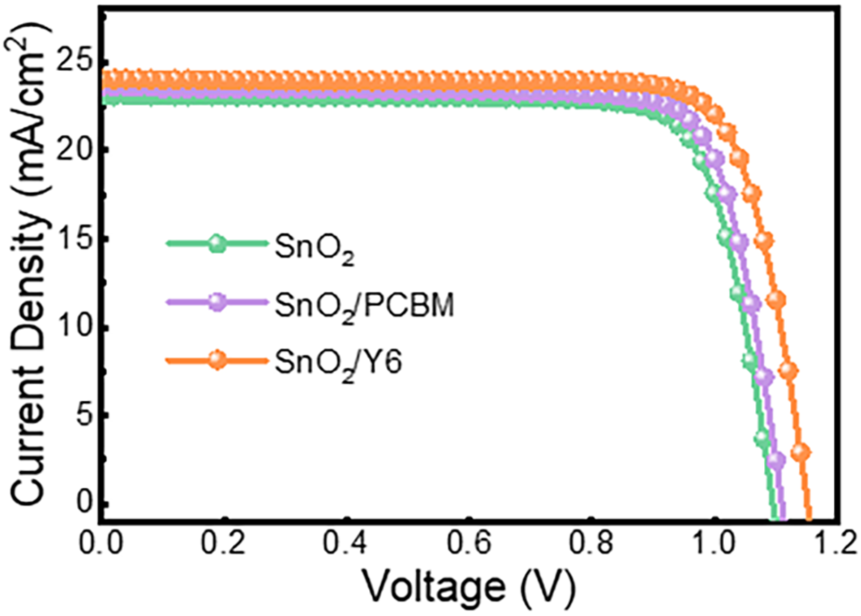

Figure S8. The *J*-*V* curves of PSCs based on SnO_2, SnO_2/PCBM and SnO_2/Y6.

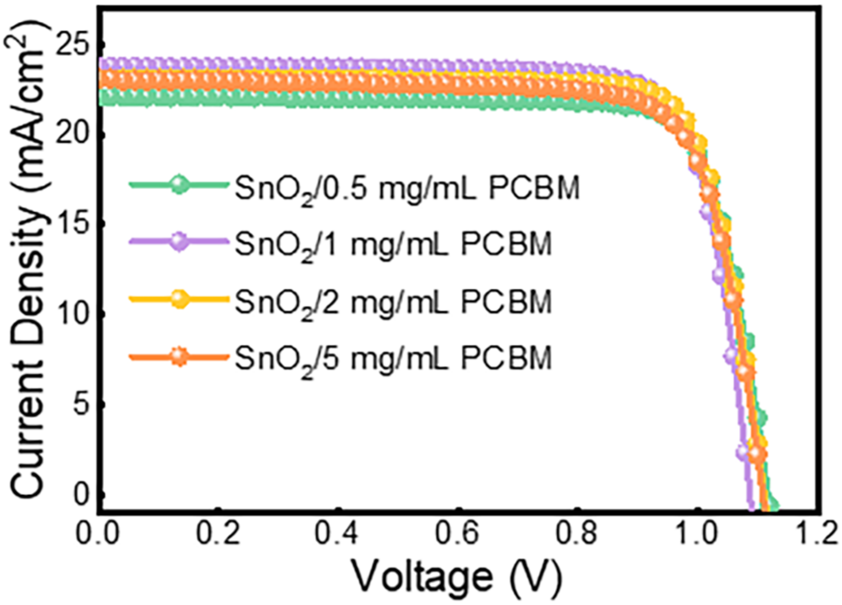

Figure S9. The *J*-*V* curves of PSCs based on different concentrations SnO_2/PCBM.

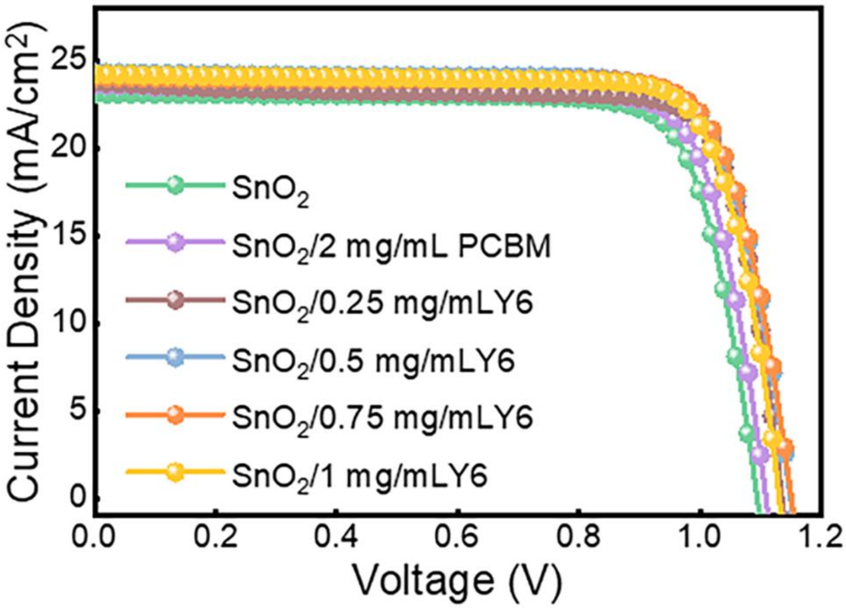

Figure S10. The *J-V* curves of PSCs based on SnO_2, SnO_2/2mg/mL PCBM and different concentrations SnO_2/Y6.

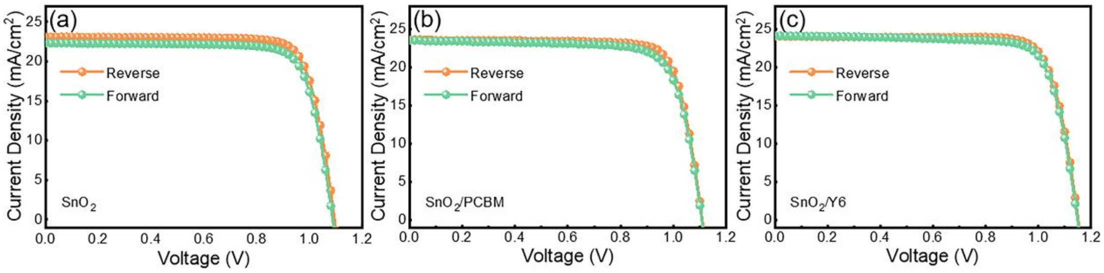

Figure S11. The reverse and forward scan *J-V* curves of PSCs based on a) SnO_2, b) SnO_2/PCBM, c) SnO_2/Y6.

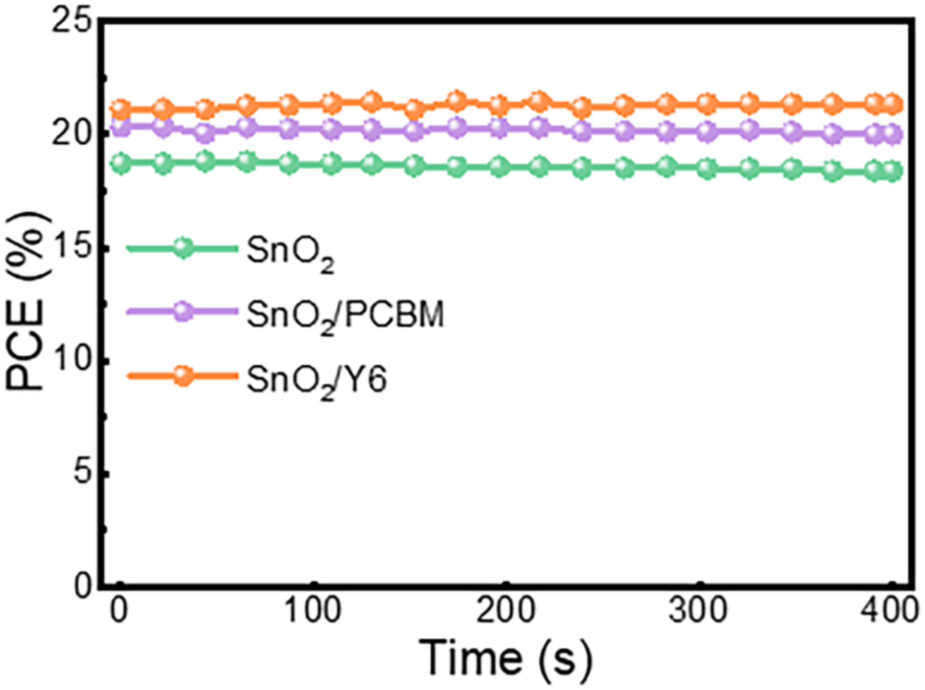

Figure S12. Steady photocurrent at max bias under 1 sun illumination.

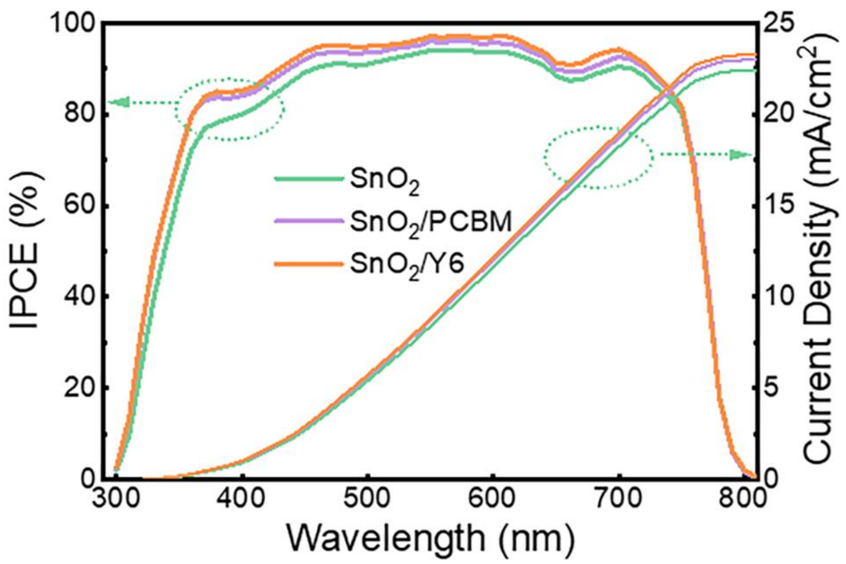

Figure S13. IPCE spectrum of SnO2/Y6-based PSCs on glasses.

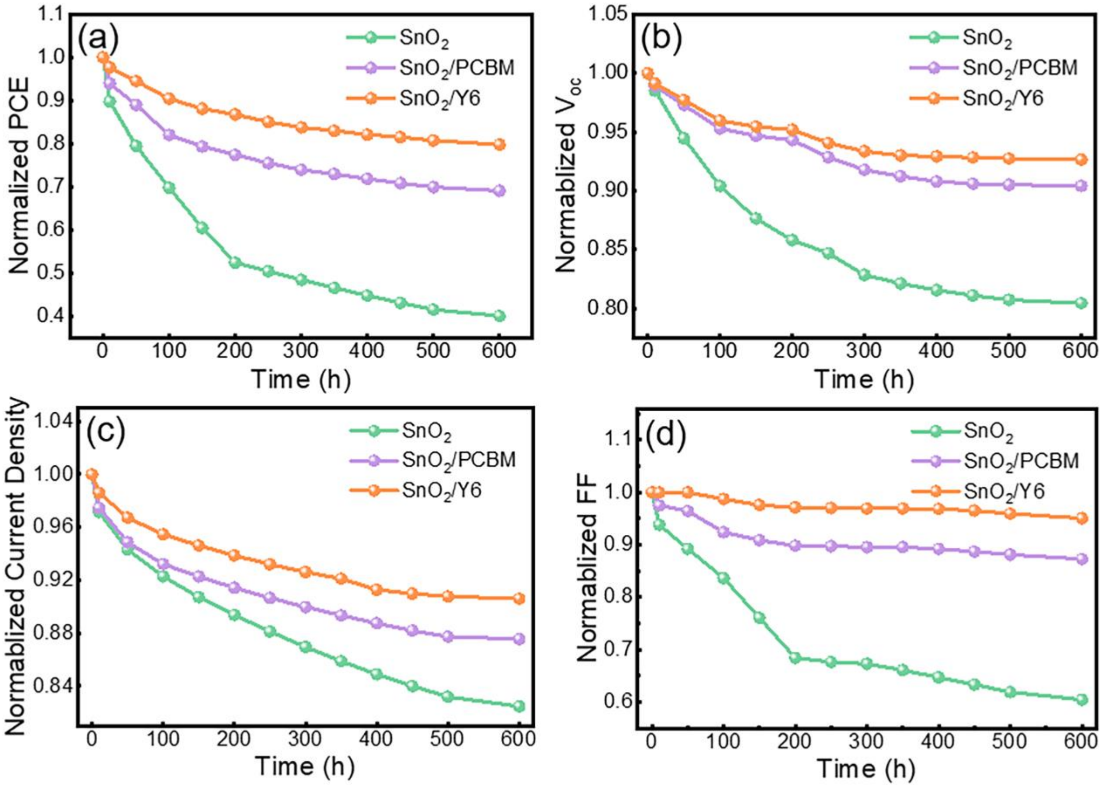

Figure S14. Long-term operational stability results of 20 cells under simulated 1-sun illumination.

TA kinetics fit

The decay kinetics was extracted at the bleaching wavelength of ~760nm. The kinetics is fitted by the following equation.

$$y = y_o + A_1 \exp\left(-\frac{t}{\tau_1}\right) + A_2 \exp(-\frac{t}{\tau_2}) \quad (1)$$

Where τ_1 and τ_2 are time constants. τ_2 is related to the excitation and decay of the charge.

TRPL fit

$$y = A_1 \exp\left(-\frac{t}{\tau_1}\right) + A_2 \exp(-\frac{t}{\tau_2}) \quad (2)$$

Where A_1 and A_2 are the amplitudes, τ_1 and τ_2 are the fast and slow decay time constants, which can directly reflect radiation recombination and trap-assisted non-radiation recombination.[1] The fitting parameters are listed in Table S2.

t-DOS fit

Trap density of states (t-DOS) can be used to characterize trap states of the complete device. To obtain the distribution of t-DOS, we measured capacitance-frequency (C_p-D) curves of these three devices and then use the equation (3) to process date of C_p-D. Where V_{bi}, q, W, C, ω, K, T are the built-in potential, elementary charge, depletion width, capacitance, angular frequency, Boltzmann constant, and temperature respectively.

$$N_t = -\frac{V_{bi}}{qW} \cdot \frac{d_C}{d_\omega} \cdot \frac{\omega}{KT} \quad (3)$$

The Mott-Schottky curve fit

$$\frac{1}{C^2} = \frac{2(V_{bi} - V)}{A^2 e \varepsilon_0 \varepsilon N_A} \quad (4)$$

The Mott-Schottky curve was tested to obtain the value of V_{bi} using the following equation.[2] where V is the applied voltage, e is the electron charge, ε_0 is the vacuum permittivity, ε is the relative dielectric constant of the inorganic perovskite, and N_A is the charge carrier concentration.

Table S1 The bleaching kinetics parameters for perovskite films grow on SnO_2 SnO_2/PCBM and SnO_2/Y6 at 760 nm

Configuration	A_1	τ_1 (ns)	A_2	τ_2 (ns)	τ_{ave} (ns)
SnO_2/PSK	2.390E-3	16.60	-1.258E-2	24.67	12.34
SnO_2/PCBM/PSK	2.412E-3	25.43	-5.932E-3	11.49	5.76
SnO_2/Y6/PSK	2.681E-3	16.30	-6.407E-3	10.09	5.05

Table S2 The fitted PL decay lifetimes of perovskite films on SnO_2, SnO_2/PCBM, and SnO_2/Y6

Configuration	A_1	τ_1 (ns)	A_2	τ_2 (ns)	τ_{ave} (ns)
SnO_2/PSK	109.30	19.45	459.42	150.46	146.52
SnO_2/PCBM/PSK	3.080	0.45	397.12	50.12	50.12
SnO_2/Y6/PSK	35.76	1.92	249.01	35.26	35.00

Table S3 Photovoltaic parameters determined from the *J-V* measurements of the FPSCs based on SnO_2, SnO_2/PCBM, SnO_2/Y6

Device	V_{OC} (V)	J_{SC} (mA/cm^2)	FF (%)	PCE (%)
PEN/ITO/SnO_2	1.08	22.4	76.9	18.60
PEN/ITO/SnO_2-300 Bending Cycles	1.01	21.7	71.9	15.76
PEN/ITO/SnO_2/Y6	1.11	22.8	79.4	20.09
PEN/ITO/SnO_2/Y6-300 Bending Cycles	1.09	22.5	76.0	18.64

Table S4 Photovoltaic parameters determined from the *J-V* measurements of the PSCs based on SnO_2, SnO_2/PCBM, SnO_2/Y6

Device	Scan Direction	V_{OC} (V)	J_{SC} (mA/cm^2)	FF (%)	PCE (%)
SnO_2	Forward	1.09	22.3	79.2	19.25
	Reverse	1.09	23.1	79.9	20.12
SnO_2/PCBM	Forward	1.11	23.5	76.9	20.06
	Reverse	1.11	23.6	80.1	20.98
SnO_2/Y6	Forward	1.15	24.1	78.4	21.73
	Reverse	1.15	24.0	80.4	22.19

Reference

[1] W. Yu, S. Yu, J. Zhang, W. Liang, X. Wang, X. Guo, C. Li, *Nano Energy*, **2018**, 18, 229.

[2] Y. Han, H. Zhao, C. Duan, S. Yang, Z. Yang, Z. Liu, S. Liu, *Adv. Funct. Mater.* **2020**, 30, 1909972.
